# Ultrafast *T*_1_–*T*_1ρ_ NMR for Correlating Different Motional Regimes of Molecules

**DOI:** 10.1021/acs.analchem.4c00513

**Published:** 2024-10-09

**Authors:** Katja Tolkkinen, Otto Mankinen, Sarah E. Mailhiot, Ville-Veikko Telkki

**Affiliations:** NMR Research Unit, Faculty of Science, University of Oulu, P.O. Box 3000, Oulu 90014, Finland

## Abstract

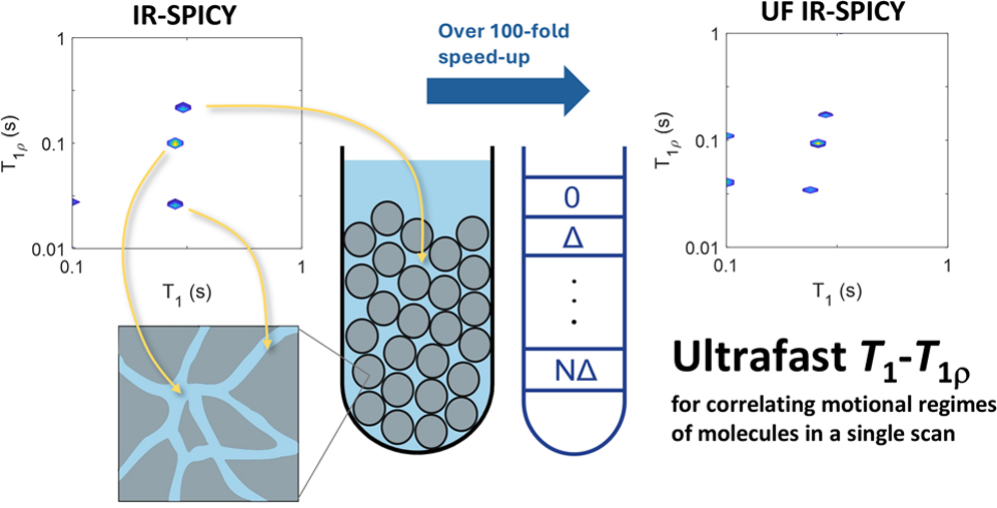

Nuclear magnetic resonance (NMR) relaxation times provide detailed information about molecular motions and local chemical environments. Longitudinal *T*_1_ relaxation time is most often sensitive to relatively fast, nano- to picosecond ranges of molecular motion. Rotating frame *T*_1ρ_ relaxation time reflects a much slower, micro- to millisecond range of motion, and the motional regime can be tuned by changing spin-lock field strength. Conventional methods for measuring *T*_1_ and *T*_1ρ_ relaxation times are time-consuming, since experiments must be repeated many times with incremented magnetization recovery or decay delay. In this work, we introduce two novel and efficient NMR methods to correlate the *T*_1_ and *T*_1ρ_ relaxation times. The first method, IR-SPICY, combines the conventional *T*_1_ inversion recovery (IR) with the single-scan *T*_1ρ_ detection-based spin-lock cycle (SPICY). The second method, ultrafast (UF) IR-SPICY, allows measurement of whole two-dimensional *T*_1_–*T*_1ρ_ correlation data in a single scan, in a couple of seconds, based on spatial encoding of the *T*_1_ dimension. We demonstrate the performance of the methods by studying relaxation of water in porous silica and hydrogel samples, latter acting as a model of the articular cartilage extracellular matrix. The methods allow correlating different molecular motional regimes, potentially providing unprecedented information about various chemical and biochemical systems, such as structures and fluid dynamics in porous materials, macromolecular changes in tissues, and protein dynamics. One to three orders of magnitude shortened experiment time enable the studies of changing or degrading samples. Furthermore, the single-scan approach may significantly facilitate the use of modern nuclear-spin hyperpolarization techniques to enhance the sensitivity of *T*_1_–*T*_1ρ_ measurements by several orders of magnitude.

Nuclear magnetic resonance (NMR) spectroscopy is a powerful technique to study chemical and physical properties of materials.^1^ Relaxation and diffusion experiments provide information on molecular dynamics and local chemical environments.^2^ These experiments result in exponentially decaying data, and the distribution of relaxation or diffusion coefficients can be extracted from the data by an inverse Laplace transform (ILT).^2^ Thus, these methods are termed Laplace NMR (LNMR). The multidimensional approach improves the resolution and information content of LNMR experiments. Two-dimensional (2D) LNMR methods correlating longitudinal relaxation time *T*_1_, transverse relaxation time *T*_2_, or diffusion coefficient *D*(3−8) have been utilized to investigate a wide variety of materials,^9^ such as biological samples,^10−12^ food products,^13−15^ rocks,^3^ geopolymers,^16^ cements,^16−18^ and shales.^17^ In addition to *T*_1_ and *T*_2_ relaxation times, *T*_2_* and *T*_1ρ_ relaxations have also been used in 2D relaxation correlation experiments.^19,20^

*T*_1_ relaxation is sensitive to molecular motions occurring close to the Larmor frequency ν_0_ = γ*B*_0_/2π, where γ is the nuclear gyromagnetic ratio and *B*_0_ is the strength of the external magnetic field.^1^ In high field instruments, the Larmor frequency is typically on the order of hundreds of megahertz. *T*_1_ relaxation times are measured by the inversion recovery (IR)^21^ or saturation recovery (SR)^22^ methods, in which the longitudinal magnetization is first inverted or destroyed, and then let to recover, and after a recovery delay read by a π/2 pulse.

*T*_1ρ_ refers to the spin–lattice relaxation in the rotating frame^23^ occurring at the presence of continuous wave (CW) spin-lock field *B*_1_. If the spin-lock pulse is applied on resonance, *T*_1ρ_ relaxation is sensitive to motions with frequencies close to the frequency of the spin-lock field ν_1_ = γ*B*_1_/2π, which is typically of the order of a few kilohertz.^24^ Therefore, *T*_1ρ_ is widely used in biomolecular research to monitor slow motions such as protein dynamics and exchange phenomena.^24,25^*T*_1ρ_ has also been broadly exploited as a contrast in magnetic resonance imaging (MRI) reflecting macromolecular changes and tissue degradation.^26^ The conventional *T*_1ρ_ experiment^27^ begins with an excitation pulse followed by a spin locking period and signal detection. A two-part spin locking with a refocusing pulse in the middle is often used to reduce effects of imperfect SL and *B*_0_ fields.^27^

Both *T*_1_ and *T*_1ρ_ experiments need to be repeated many times with an incremented recovery or spin-lock delay to follow the magnetization recovery or decay process. This leads to rather long experiment times. Long experiment times restrict the investigation of systems, which are rapidly changing or degrading. Furthermore, the need for repetitions makes it difficult to use nuclear-spin hyperpolarization techniques, such as dynamic nuclear polarization,^28^ parahydrogen-induced polarization,^29,30^ and spin-exchange optical pumping,^31^ for improving the sensitivity of experiments by several orders of magnitude.

Ultrafast (UF) NMR spectroscopy^32−35^ offers a means to collect 2D data in a single scan. The method is based on the spatial encoding of evolution times in the layers of a sample. Spatial encoding has also been used for single-scan measurements of *T*_1_ IR data.^36^ We have exploited the principles of spatial encoding to develop a multitude of UF relaxation and diffusion correlation and exchange experiments.^37−42^ Furthermore, we have shown that these single-scan UF LNMR experiments can be combined with hyperpolarization,^37−45^ which enables, for example, the tracking of metabolites in intracellular and extracellular spaces.^46^

Articular cartilage is a dense avascular tissue covering the ends of the bones.^47^ It is made up of chondrocytes surrounded by the extracellular matrix, which mainly contains water, collagen, and proteoglycans.^48^ Proteoglycans are macromolecules containing a protein core with attached glycosaminoglycan (GAG) chains, and they tend to interact with hyaluronan to form large proteoglycan aggregates. The GAGs present in articular cartilage are chondroitin sulfate (CS) and keratin sulfate (KS), of which CS is present in larger amounts. The GAG chains provide cartilage tissue its osmotic properties and ability to resist compressive loads.^47,48^

The loss of proteoglycans is believed to be the initial event in the formation of osteoarthritis, a degenerative disease of articular cartilage.^49^ Rotating frame relaxometry has been extensively used to investigate the different conditions of cartilage degradation and osteoarthritis.^50−53^ Previous research show that *T*_1ρ_ relaxation is more sensitive to the proteoglycan content in cartilage than other relaxation methods,^54−56^ which makes *T*_1ρ_ MRI an important tool in the detection of early osteoarthritis. Water molecules in cartilage extracellular space can be located in the bulk water or be bound to protein compartments, which leads to multicomponent *T*_2_ and *T*_1ρ_ relaxations;^10,57−60^ however, *T*_1_ relaxation has been shown to have only one component.^10^

Recently, we introduced a novel method, called spin-lock cycle (SPICY),^61^ for the measurement of *T*_1ρ_ relaxation times in a single scan. In the SPICY sequence (Figure 1a), after a single excitation pulse, the spin locking and echo detection are repeated with a loop, which allows determination of the *T*_1ρ_ times in only one scan by fitting the exponential decays to the echo intensities corresponding to different echo numbers. This approach reduces the *T*_1ρ_ measurement time by an order of magnitude compared to the conventional *T*_1ρ_ sequence,^32^ which must be repeated with incremented CW spin-lock pulse durations. We also introduced a single-scan 1D imaging variant of SPICY, which broadens its applicability toward MRI applications.

**Figure 1:**
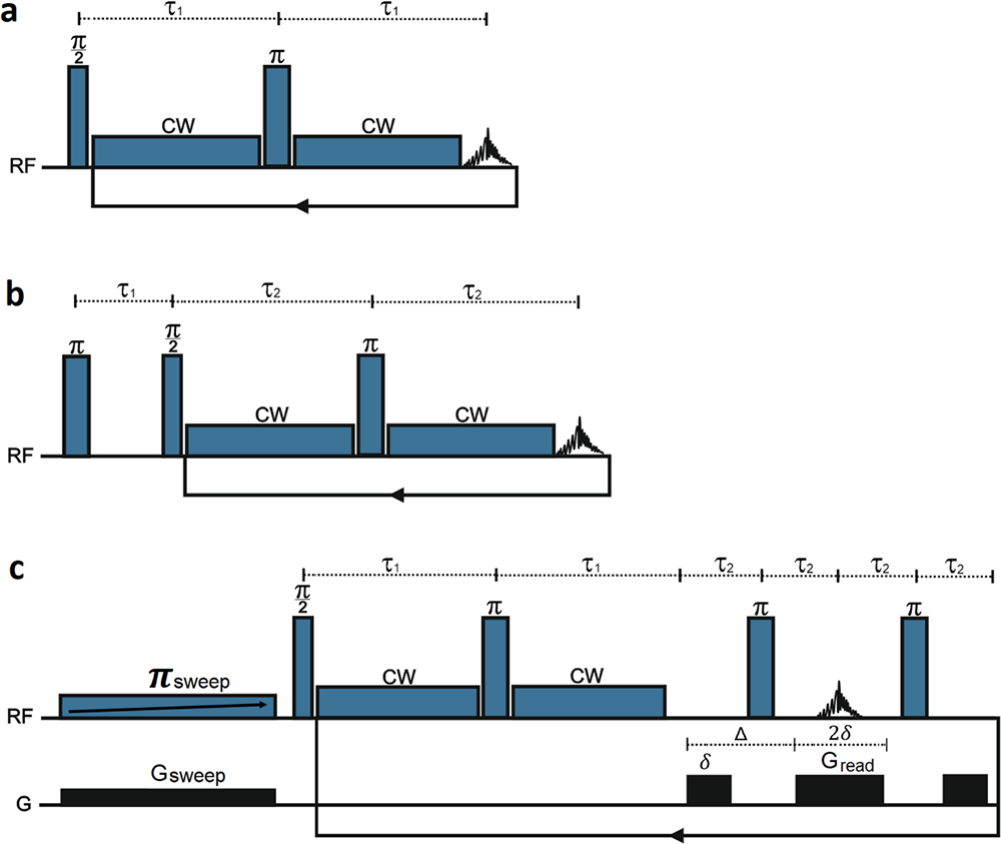
Pulse sequences for (a) SPICY, (b) IR-SPICY, and (c) UF IR-SPICY experiments.

Here, we introduce a novel and efficient *T*_1_–*T*_1ρ_ correlation experiment called IR-SPICY, which combines the conventional IR *T*_1_ encoding part with single-scan SPICY detection. Since *T*_1_ and *T*_1ρ_ probe high- and low-frequency motions, respectively, IR-SPICY measures the correlation of two types of molecular motions. In addition, based on spatial encoding, we introduce a novel UF IR-SPICY experiment, which makes it possible to collect full 2D *T*_1_–*T*_1ρ_ data in a single scan, in a few seconds. We demonstrate the performance of these methods by investigating the relaxation of water in porous silica gel and collagen–CS hydrogel samples. Protein hydrogels have been extensively used in many biomedical applications, such as in tissue engineering.^62^ In this work, the hydrogel represents a simple model of the structure of the cartilage extracellular matrix. To the best of our knowledge, 2D *T*_1_–*T*_1ρ_ correlation measurements have not been described in the literature earlier.

## Experimental Section

### Sample Preparation

The porous material sample included silica gel (Merck, Darmstadt, Germany) powder with the mean pore diameter of 6 nm and particle size of 63–200 μm immersed in H_2_O in a 5 mm NMR tube.

The hydrogel sample contained 40 mg/g collagen type II, 10 mg/g CS, and phosphate-buffered saline (PBS). The gel was prepared by adding 10 mL of collagen solution (rat tail, ∼6 mg/mL in 0.01 M acetic acid, Merck KGaA, Darmstadt, Germany) to a sample tube together with 0.15 mL of 10× PBS. Stock solution of CS (50–80 mg/mL CS powder from bovine trachea, Merck KGaA, Darmstadt, Germany, in double-distilled water) was added to the collagen solution, and the mixture was diluted with double-distilled water to give a total volume of 6 mL. The sample was thoroughly mixed, and pH was adjusted to basic (pH: 8–9) using 1 M sodium hydroxide (NaOH) and 0.5 M hydrochloric acid (HCl). The sample tube was placed for 45–60 min in a water bath at 37 °C to solidify the mixture into a gel. The solid was removed from the sample tube, transferred to a plastic cell culture dish, and dried at 40 °C in a laminar flow oven until the mass of the sample was reduced to 1.50 ± 0.03 g in order to achieve final collagen concentrations higher than the concentration of the commercially obtained solution. Finally, the sample was placed in a 5 mm NMR tube.

### NMR Experiments

^1^H NMR experiments were performed with a Bruker Avance III 600 MHz NMR spectrometer using a quadruple-resonance (QXI) probe. The lengths of hard π/2 and π pulses were 10 and 20 μs for the silica gel and 13 and 26 μs for the hydrogel samples, respectively. The data analysis including peak integration, curve fitting, and spin density and coil sensitivity profile correction^63^ was performed using MATLAB (MathWorks, R2020b, Natick, Massachusetts, USA). The 1D relaxation time distributions and 2D correlation maps were computed by the ITAMeD^64^ ILT program, which uses the non-negativity constraint and Tikhonov regularization based on the *l*_1_-norm to make the ILT stable and does not require any *a priori* knowledge about the number of relaxation components. From the several solutions that are consistent with experimental data, ITAMeD chooses one with the smallest number of components.^64^

#### 1D Relaxation Experiments

*T*_2_ relaxation times were determined with the CPMG^65^ pulse sequence and *T*_1_ relaxation times with the IR pulse sequence.^21^ In the CPMG measurement of the silica gel sample, the number of echoes was 2000, echo time 200 μs, number of scans 8, and relaxation delay 5 s. Corresponding values for the hydrogel sample were 2000 echoes, 200 μs echo time, 8 scans, and 15 s recovery delay. In the IR experiment of the silica sample, the recovery delay τ was incremented between 1 ms and 5 s in 50 steps, and in the hydrogel experiment between 1 ms and 15 s in 50 steps. *T*_1ρ_ relaxation times were measured with both the conventional *T*_1ρ_ method^27^ and SPICY (Figure 1a).^61^ In the conventional *T*_1ρ_ sequence, the CW spin-lock pulse length was incremented between 10 and 450 ms in 50 steps. In the SPICY sequence, the constant CW spin-lock pulse length was 6 ms, number of echoes *n* = 64, the total signal detection time 0.77 s, and the CW pulse off time during the echo acquisition 100 μs. Twenty-five data points were collected at each echo. In the *T*_1ρ_ experiments, the spin-lock frequency was 7 kHz for the silica sample and 5 kHz for the hydrogel sample. The CW spin-lock pulses were applied on resonance. The number of scans was 8 in all of the experiments. The relaxation delay was 5 s in the silica gel experiments and 15 s in the hydrogel experiments.

In the ILT analysis of the silica gel data, the parameters for the IR data were 1,000,000 iterations with inversion limits between 0.01 and 10 s, and for the *T*_1ρ_ data, 500,000 iterations and inversion limits 0.01 and 1 s. Correspondingly for the hydrogel, the parameters in the IR data analysis were 10,000 iterations with inversion limits between 0.1 and 10 s, and in the *T*_1ρ_ analysis, 5,000,000 iterations with inversion limits 0.01 and 1 s.

#### IR-SPICY

The IR-SPICY sequence consists of consecutive IR and SPICY blocks (Figure 1b). The measurement parameters in the IR and SPICY parts were identical to the parameters used in the reference IR and SPICY measurements, leading to collection of data matrices with a size of 50 × 64. The number of accumulated scans was 8. In the silica gel measurement, the relaxation delay was 5 s and the total experiment time 50 min 11 s, and in the hydrogel measurement, the corresponding values were 15 s and 2 h 33 min, respectively. The 15 s relaxation time was used for the hydrogel to prevent the gel from melting.

The ILT for the silica gel data was implemented with 500,000 iterations, grid size of 30 × 100, and inversion limits of 0.1–10 s in the *T*_1_ direction and 0.01–1 s in the *T*_1ρ_ direction. Corresponding values for the ILT of the hydrogel data were 2,000,000 iterations, grid size of 30 × 100, and inversion limits 0.1–10 s in the *T*_1_ direction and 0.01–1 s in the *T*_1ρ_ direction.

#### UF IR-SPICY

The UF IR-SPICY sequence (Figure 1c) includes an adiabatic frequency-swept chirp inversion pulse applied simultaneously with a gradient pulse, followed by the SPICY imaging block. The length of the adiabatic π chirp inversion pulse was 2 s for the silica gel and 5 s for the hydrogel, the chirp pulse bandwidth was 10 kHz, and the spatial encoding gradient strength *G*_s_ was 30 mT m^–1^. In the SPICY block, the number of echoes was 32, CW pulse duration 12 ms, the read gradient duration 2.56 ms, read gradient strength *G*_read_ 84 mT m^–1^, and the sweep width 100 kHz. Five hundred twelve data points were acquired at each echo with a dwell time of 5 μs. The gradients had a trapezoidal shape with a gradient ramp delay of 50 μs. The CW off time in a single loop (4τ_2_ in Figure 1c) was 8 ms. The spin-lock frequency for silica gel was 7 kHz and that for hydrogel 5 kHz. The number of scans was one, and the experiment time was 3 s in the silica gel experiment and 6 s in the hydrogel experiment. The 1D spin echo image for the coil profile correction was acquired with 32 echoes, 24 ms echo time, and 84 mT m^–1^ read gradient strength.

The ILT parameters for the UF IR-SPICY data of silica gel and hydrogel samples were 500,000 iterations, grid size 30 × 100, and inversion limits 0.1–10 s in the *T*_1_ direction and 0.01–1 s in the *T*_1ρ_ direction.

## Results and Discussion

### Novel and Efficient *T*_1_–*T*_1ρ_ Correlation Experiments

The pulse sequence for the IR-SPICY *T*_1_–*T*_1ρ_ correlation measurement is shown in Figure 1b. The sequence starts with a hard π pulse that inverts the longitudinal magnetization. Thereafter, the longitudinal magnetization is let to recover toward the thermal equilibrium due to *T*_1_ relaxation for the delay τ_1_. The following hard π/2 pulse rotates longitudinal magnetization to the transverse plane, where it is locked along the *y*-axis by the CW pulse with a spin-lock field of *B*_1_. The locking is interrupted by the π refocusing pulse and the short period for signal acquisition. Because only the detection of the echo center provides the points for *T*_1ρ_ decay fitting in the SPICY sequence, the SL gap due to echo detection can be set short and the contribution of *T*_2_ is negligible in the IR-SPICY sequence. The spin-lock and signal acquisition period are looped *N*_T1ρ_ times to collect the *T*_1ρ_ decay data in a single scan. This leads to *N*_T1ρ_-fold reduction in the experiment time as compared to the conventional approach, in which *T*_1ρ_ decay data are collected in repeated experiments with an incremented spin-lock period. The IR-SPICY sequence needs to be repeated *N*_T1_ times with an incremented τ_1_ delay for collecting *T*_1_ recovery data. Typically, *N*_T1_ and *N*_T1ρ_ values vary from 8 to 64. The signal observed in the IR-SPICY experiment can be described by the equation
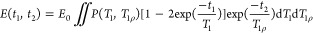
1where *P*(*T*_1_, *T*_1ρ_) is the 2D distribution of relaxation times *T*_1_ and *T*_1ρ_. The 2D ILT can be used to resolve the relaxation time distribution from the experimental data.

The IR-SPICY sequence can be further accelerated by spatial encoding of the *T*_1_ dimension, as illustrated in Figure 1c. This UF IR-SPICY sequence begins with an adiabatic π_sweep_ frequency–swept chirp inversion pulse, with its frequency linearly increasing with time. As the π_sweep_ pulse is applied simultaneously with the gradient *G*_sweep_, which makes the Larmor frequencies of spins linearly dependent on position, the spins at the bottom of the spatial encoding region are inverted first and those at the top last. Therefore, the magnetization recovery time between the inversion moment and the hard π/2 pulse becomes linearly dependent on position, being zero at the top and equal to the π_sweep_ pulse length at the bottom, and the longitudinal magnetization profile from top to bottom becomes equivalent to the conventional exponentially increasing IR curve. This profile is read, with the read gradients added to the SPICY loop. Since the signal acquisition part includes gradients, the spin-lock interruption is longer compared to the spin-lock gap due to echo acquisition in the IR-SPICY sequence. The duration of the signal readout period is 4τ_2_ (Figure 1c), and during this period, *T*_2_ relaxation and molecular diffusion affect the signal decay. The signal decay as a function of echo number *n* can be expressed as
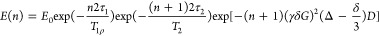
2where δ is the gradient duration (half the length of the read gradient), *G* the read gradient strength, Δ the diffusion time (the time from the beginning of the dephasing gradient to the beginning of the read gradient), and *D* the diffusion coefficient. The first exponential term in eq 2 represents *T*_1ρ_ decay during spin locking, the second term *T*_2_ decay during the signal readout period, and the third term decay due to the combined effect of molecular diffusion and read gradient pulses. In the case of single-component relaxation, the fit of eq 2 with the experimental signal decay gives the correct *T*_1ρ_ value, if *T*_2_ and *D* values are fixed to the values determined in separate relaxation and diffusion experiments. If the spin lock gap is made as short as possible, in many practical applications eq 1 gives an adequate model for data analysis in the case of multicomponent relaxation. Here, 2D ILT analysis was performed by using eq 1.

The UF IR-SPICY experiment enables measurement of 2D *T*_1_–*T*_1ρ_ correlation data in a single scan, leading to *N*_T1_-fold (typically 8–64-fold) reduced experiment time as compared to the IR-SPICY experiment, in which the time variable corresponding to the *T*_1_ dimension is incremented in repeated experiments, and *N*_T1_ × *N*_T1ρ_-fold (typically 64–4096-fold) reduced experiment time as compared to the *T*_1_–*T*_1ρ_ correlation experiment performed with the conventional approach, in which time variables corresponding to both *T*_1_ and *T*_1ρ_ dimensions are incremented in repeated experiments.

### Relaxation Components in the Porous Material Sample

**Figure 2:**
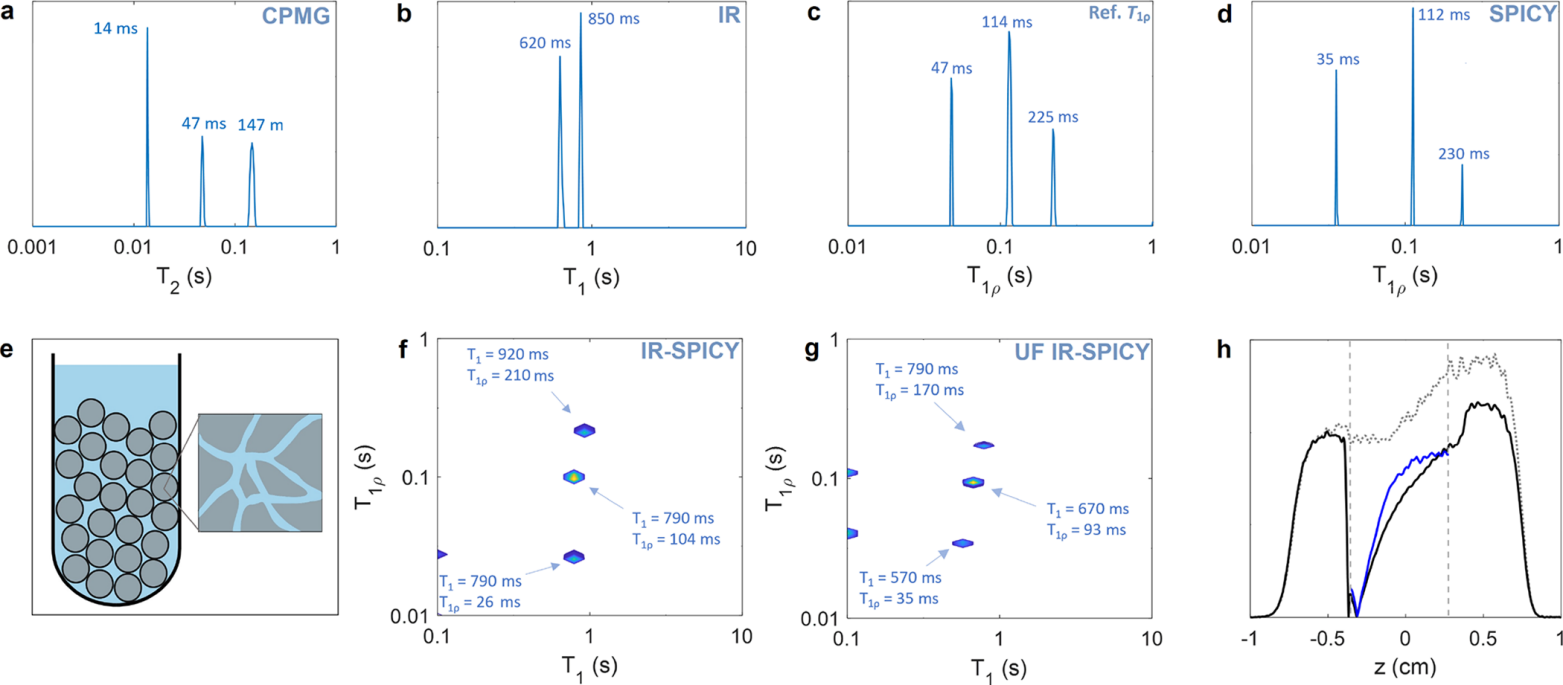
^1^H *T*_2_, *T*_1_, and *T*_1ρ_ relaxation time distributions measured for silica gel sample with (a) CPMG, (b) conventional IR, (c) conventional *T*_1ρ_, and (d) SPICY. (e) Illustration of the sample including porous silica gel powder (pore diameter 6 nm; particle size 63–200 μm) immersed in water. 2D ^1^H *T*_1_–*T*_1ρ_ correlation maps obtained with (f) the IR-SPICY and (g) UF IR-SPICY methods. The latter was measured in a single scan with an experiment time of only 3 s. The marked values indicate the peak centers. (h) 1D magnetization profiles measured with the UF IR-SPICY sequence (black solid line, first echo) and the 1D spin echo imaging sequence (gray dotted line). The latter was used in the spin density and coil sensitivity profile correction to obtain the corrected magnetization profile shown by the blue solid line. Vertical dashed lines show the data range that was used in the ILT analysis.

The feasibility and reliability of the proposed new *T*_1_–*T*_1ρ_ correlation experiments were first demonstrated by studying relaxation of ^1^H nuclei of water molecules in the sample including porous silica gel powder immersed in water (Figure 2e). The 1D relaxation time distributions obtained by the conventional *T*_1_ IR and *T*_1ρ_ methods show that the silica sample possesses two separate *T*_1_ components at 620 ± 40 and 850 ± 30 ms and three *T*_1ρ_ components at 47 ± 2, 114 ± 8, and 225 ± 14 ms (Figure 2b,c). The error values are the larger distances from the peak maximum to the zero point on the left and right, reflecting the line width. The shortest and longest *T*_1ρ_ components originate most likely from water located in the 6 nm-sized pores and from the bulk-like water between the 63 and 200 μm-sized particles,^66^ and the intermediate component may be a result of relatively fast exchange of water molecules close to the surface of particles between these two sites.^37^ The *T*_1ρ_ values measured by SPICY (35 ± 2, 112 ± 5, and 230 ± 8 ms, Figure 2d) are otherwise in agreement with the values obtained by the conventional *T*_1ρ_ measurement (47 ± 2, 114 ± 8, and 225 ± 14 ms) within error bars, except that the *T*_1ρ_ of the first component is underestimated by about 25%. The deviation is most probably a consequence of the limited number of points for probing the shortest *T*_1ρ_ component, as the echo time in the experiments (10–12 ms) was comparable to that of the shortest *T*_1ρ_ (35–47 ms). The *T*_2_ relaxation time distribution measured by CPMG (Figure 2a) also includes three components at 14 ± 1, 47 ± 4, and 147 ± 18 ms. As expected, the *T*_1ρ_ values fall between the *T*_2_ and *T*_1_ values.

The *T*_1_–*T*_1ρ_ relaxation correlation map measured with the IR-SPICY (Figure 2f) shows three components with *T*_1_ values of 790 ± 140 and 920 ± 160 ms, and *T*_1ρ_ values of 26 ± 5, 104 ± 10, and 210 ± 40 ms. The number of components and measured relaxation time values are in good agreement with the 1D reference measurements within error bars, except the shortest *T*_1ρ_ value, which was about a half shorter than in the conventional *T*_1ρ_ measurement, again most probably due to the limited sampling of the fast-decaying component. The IR-SPICY data was measured with eight accumulated scans, and it included 50 τ_1_ increments in repeated experiments. The total experiment time was 50 min. The SPICY detection block allowed the measurement of all 64 data points of the *T*_1ρ_ dimension in a single scan, resulting in 64-fold reduction in the experiment time as compared to a similar experiment performed with the conventional approach. In such a hypothetical identical conventional experiment, the experiment time would be 53 h instead of 50 min.

The *T*_1_–*T*_1ρ_ map obtained with UF IR-SPICY (Figure 2g) also includes three components similar to the IR-SPICY map. The *T*_1ρ_ values (35 ± 4, 93 ± 9, and 170 ± 20 ms) obtained with UF IR-SPICY are shortened from the values given by the 1D reference measurements about 18–25% due to the molecular diffusion in the presence of the read gradients in the SPICY loop as well as longer interruption in the spin locking. The instability of the Laplace inversion and sensitivity to experimental noise may also affect the obtained values (see discussion below).^61^ According to the 1D spin echo image (gray dotted line in Figure 2h), the spin density increases toward positive *z* values due to the inhomogeneity of the sample, affecting the spatial encoding profile in the UF IR-SPICY experiment (black solid line in Figure 2h). This effect was compensated by dividing the UF IR-SPICY spatial encoding profiles by the 1D spin echo image. Resulting compensated spatial encoding profile is shown by the blue solid line in Figure 2h. The *T*_1_ values (570 ± 100, 670 ± 120, and 790 ± 140 ms) observed in the UF IR-SPICY experiments are in agreement with the 1D reference experiment within error bars. Some typical ILT edge artifacts due to experimental noise are seen on the left side of both the IR-SPICY and UF IR-SPICY maps. Remarkably, the duration of the 2D UF IR-SPICY experiment performed by a single scan was 3 s. As the UF IR-SPICY data extracted for the 2D ILT included 60 points in the *T*_1_ dimension and 32 points in the *T*_1ρ_ dimension, hypothetical similar experiments performed with the IR-SPICY and the conventional approach would have 60 and 60 × 32 = 1920 times longer experiment times (3 min and 1.6 h instead of 3 s), respectively.

### Relaxation Components in the Hydrogel Sample

**Figure 3:**
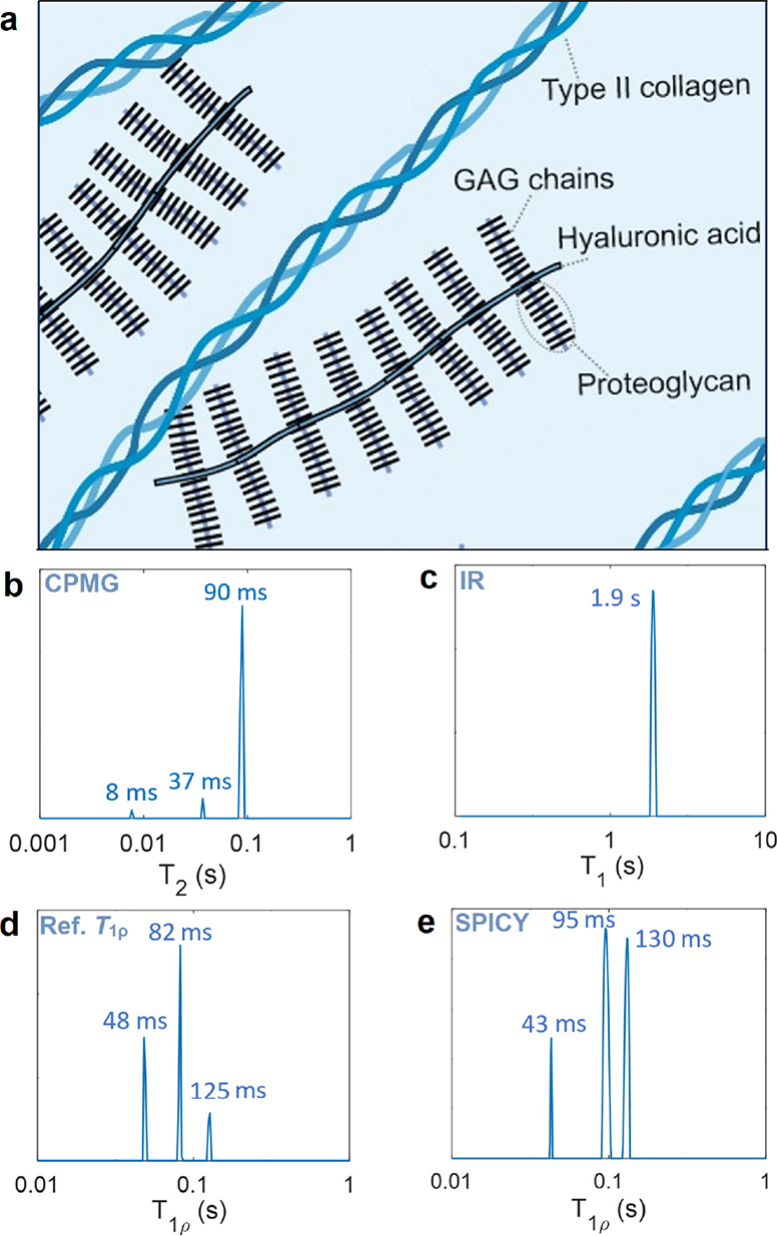
(a) Illustration of cartilage extracellular matrix. The hydrogel in this study contains type II collagen and GAG chains. 1D *T*_2_, *T*_1_, and *T*_1ρ_ relaxation time distributions of the hydrogel sample measured with (b) CPMG, (c) inversion recovery, (d) conventional *T*_1ρ_ sequence, and (e) SPICY. The marked values indicate the peak centers.

In the second demonstration, the proposed new *T*_1_–*T*_1ρ_ correlation experiments were used to study relaxation of ^1^H nuclei of water molecules in a collagen–CS hydrogel sample resembling the protein content of the articular cartilage extracellular matrix (Figure 3a). The 1D relaxation time distributions show that the hydrogel sample possesses three *T*_2_ components at 8 ± 1, 37 ± 2, and 90 ± 8 ms, three *T*_1ρ_ components at 48 ± 3, 82 ± 4, and 125 ± 6 ms and a single *T*_1_ component at 1.9 ± 0.1 s (Figure 3b–e). The *T*_1ρ_ values given by SPICY are 43 ± 2, 95 ± 9, and 130 ± 9 ms. The relaxation time distributions measured by the conventional *T*_1ρ_ and SPICY methods are in good agreement within error bars, although the peak intensities differ somewhat due to the challenges of the ILT to resolve components close to each other.^63^ The three *T*_1ρ_ components may arise from H_2_O molecules in bulk water and attached to collagen and CS. This is in agreement with the previous studies, which showed that cartilage tissue contains two or three *T*_1ρ_ components which originate from bulk as well as collagen and proteoglycan-bound water.^10,57−60^ A single *T*_1_ could imply that exchange between the water pools is fast in the *T*_1_ time scale, which is much longer (1.9 s) than *T*_2_ and *T*_1ρ_ time scales (8–130 ms).^10^

**Figure 4:**
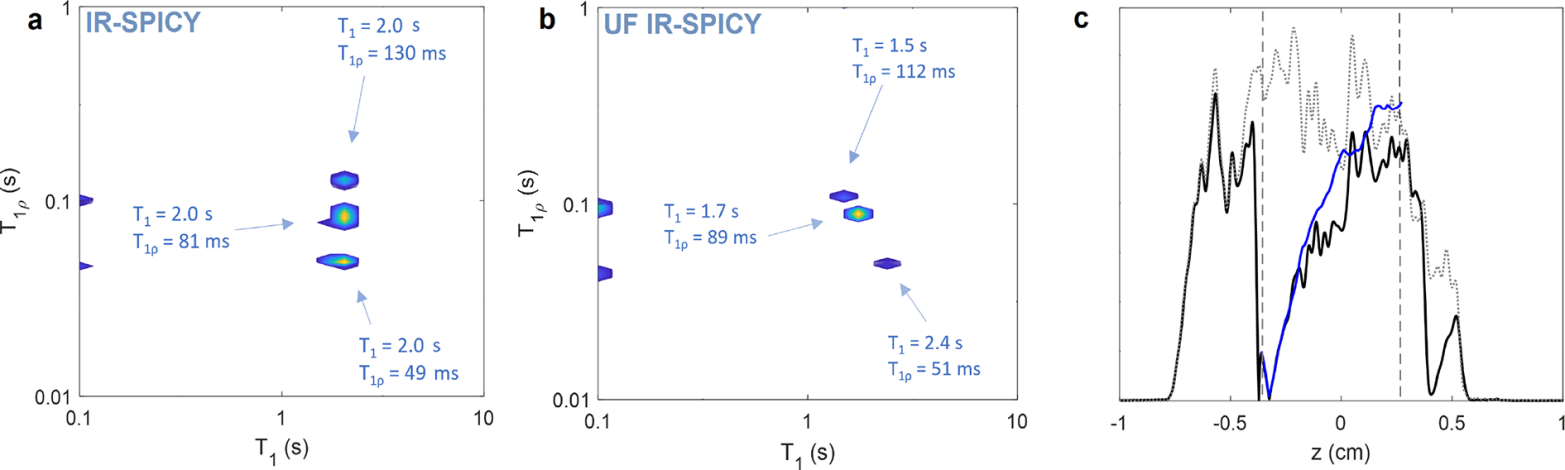
^1^H *T*_1_–*T*_1ρ_ correlation maps of the hydrogel sample measured by (a) IR-SPICY and (b) UF IR-SPICY methods. The marked values indicate the peak centers. (c) 1D magnetization profiles measured with the UF IR-SPICY sequence (black solid line, first echo) and the 1D spin echo imaging sequence (gray dotted line). The latter was used in the spin density and coil sensitivity profile correction to obtain the corrected magnetization profile shown by the blue solid line. Vertical dashed lines show the data range, which was used in the analysis.

The 2D *T*_1_–*T*_1ρ_ relaxation correlation maps measured by the IR-SPICY (Figure 4a) show three separate peaks with *T*_1ρ_ of 49 ± 5, 81 ± 15, and 130 ± 17 ms and T_1_ of 2.0 ± 0.5 s. The values are in good agreement with the values measured in the 1D reference measurements within the error bars. The duration of the IR-SPICY experiment was 2.5 h with eight scans and 50 τ_1_ increments.

The UF IR-SPICY *T*_1_–*T*_1ρ_ correlation maps also include three peaks. Measured *T*_1ρ_ values (51 ± 5, 89 ± 9, and 112 ± 10 ms) are in good agreement with those obtained by the 1D reference measurement. The 1D spin echo image of the sample (gray dotted line in Figure 4c) shows that the spin density in the sample is inhomogeneous due to air gaps in the gel. Again, the UF IR-SPICY spatial encoding profile (black solid line in Figure 4c) was corrected by using the 1D spin echo image before the ILT analysis. However, some irregularities remained in the spatial encoding profile, even after the correction (blue solid line in Figure 4c). Contrary to the other experiments, the *T*_1_ values (1.5 ± 0.3, 1.7 ± 0.3, and 2.4 ± 0.4 s) in the UF IR-SPICY *T*_1_–*T*_1ρ_ correlation maps are not equal, which is most likely a small artifact originating from the remaining irregularities in the spatial encoding profile. The duration of the single-scan UF-SPICY experiment was only 6 s.

### Comparison of the *T*_1_–*T*_1ρ_ Correlation Experiments

An essential difference between IR-SPICY and UF IR-SPICY is the overall duration of the CW spin-lock pulse compared to the total signal detection time in the SPICY loop. In the IR-SPICY, the CW pulse off time during the acquisition period can be reduced to as short as tens or hundreds of microseconds because only a few data points need to be detected around the echo center. When the acquisition part is short, almost the entire relaxation takes place under the influence of spin locking. Therefore, the signal decays almost purely due to *T*_1ρ_, as evidenced by the good agreement between the *T*_1ρ_ values observed in the IR-SPICY and conventional *T*_1ρ_ experiments described above within error bars, except for the improperly probed short *T*_1ρ_ component in the case of the silica sample.

**Figure 5:**
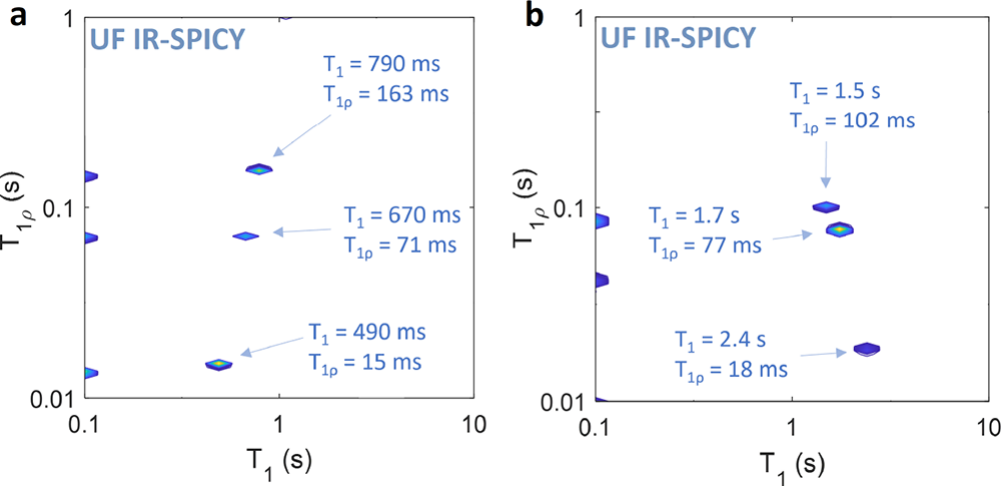
^1^H *T*_1_–*T*_1ρ_ correlation maps measured by UF IR-SPICY with 64 echoes for (a) silica sample and (b) hydrogel sample.

In the UF IR-SPICY, it is not possible to obtain as efficient spin locking because the acquisition period is longer due to the imaging gradients with associated gradient stabilization delays and greater number of points collected for full 1D image data. During the acquisition period, signal decays faster due to *T*_2_ and molecular diffusion in the presence of the read gradients according to eq 2.^6,63^ The CW spin-lock pulse on/off ratio can be increased by shortening the gradients and data acquisition, increasing the CW pulse length and reducing the number of collected echoes. On the other hand, reducing the number of points reduces resolution of the experiment and makes it more difficult to resolve multiple relaxation components in the ILT procedure. In the UF IR-SPICY experiment described above, the CW pulse was applied 75% of the total signal detection time, leading to apparent *T*_1ρ_ values that differ approximately 6–25% from the reference values. The UF IR-SPICY sequence was additionally tested with both samples using a double number of echoes (64) and shorter CW pulse duration (6 ms), in which case the CW pulse was applied only 60% of the total signal detection time. With higher number of echoes and shorter spin locking time, the *T*_1_ values did not remarkably change but the measured *T*_1ρ_ values were systemically decreased compared to the values obtained with 32 echoes and 12 ms CW pulse duration (Figure 5). Although it is challenging to observe fully quantitative *T*_1ρ_ values in the UF IR-SPICY experiments, in many applications, it is sufficient to maintain intercomponent resolution, as it was possible in the porous material and hydrogel experiments described above.

**Figure 6:**
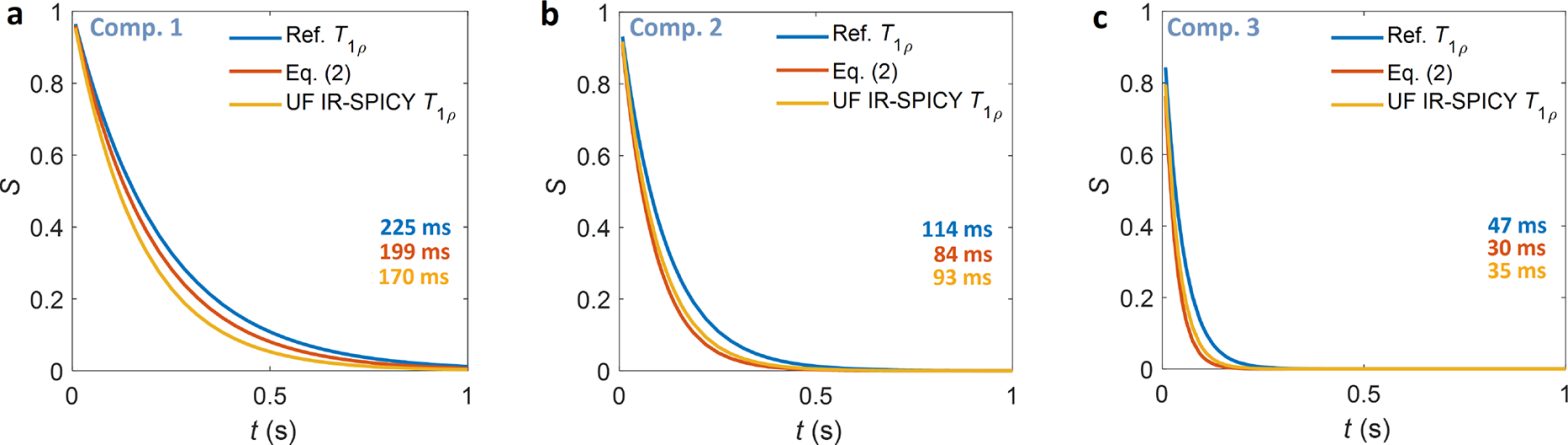
Modeled *T*_1ρ_ decays for the (a) longest, (b) middle, and (c) shortest *T*_1ρ_ components of the silica gel sample. The blue and yellow curves were calculated by the equation *S* = exp (− t/*T*_1ρ_) using the *T*_1ρ_ values measured with the conventional *T*_1ρ_ method (225, 114, and 47 ms) and UF-IR-SPICY (170, 93, and 35 ms). The red curves represent the effective *T*_1ρ_ decay calculated using eq 2 using the *T*_1ρ_, *T*_2_, and *D* values obtained from reference experiments, and the effective *T*_1ρ_ rates are shown by the red numbers (199, 84, and 30 ms). The simulation using eq 2 gives effective *T*_1ρ_ rates that are shortened compared to the reference *T*_1ρ_ values and closer to the values measured with the UF IR-SPICY method.

We simulated the accelerated signal decay in the UF IR-SPICY experiments using eq 2. Figure 6 illustrates modeled *T*_1ρ_ signal decays for three relaxation time components of the silica sample. Exponential decays are plotted using *T*_1ρ_ values obtained with the conventional *T*_1ρ_ method and UF IR-SPICY method and using the *T*_2_ and diffusion-weighted *T*_1ρ_ decay obtained with eq 2. The diffusion coefficient *D* 1.9 × 10^–9^ m^2^ s^–1^ was taken from our previous study.^42^Equation 2 gives effective decay constants (30, 84, and 199 ms), which are close to the apparent *T*_1ρ_ values obtained with UF IR-SPICY (35, 93, and 170 ms), showing that eq 2 describes well the effect of *T*_2_ and *D* on signal decay due to the to the SL interruptions in the UF-IR-SPICY experiments.

The IR-SPICY experiment time was 50 min in the silica gel measurement and 2.5 h in the hydrogel measurement. Eight scans were accumulated to execute the eight-step phase cycle. However, the IR-SPICY works also sufficiently without phase cycling. As described above, the IR-SPICY is *N*_T1_ times (in this case, *N*_T1_ = 50) more efficient than a hypothetical conventional *T*_1_–*T*_1ρ_ correlation experiment. The acceleration due to the SPICY loop occurs without any sensitivity penalty. However, as only a few data points are collected in each SPICY loop, the IR-SPICY experiment lacks a chemical shift resolution. If needed, partial chemical shift resolution can be achieved, for example, by frequency-selective pulses.^63^

The UF IR-SPICY was performed with only one scan, and the experiment time was only a few seconds. The experiment time was reduced by the factors of *N*_T1_ and *N*_T1_ × *N*_T1ρ_ (in this case, 60 and 1920) compared to the identical IR-SPICY and conventional approaches, respectively. The price to pay for the significant acceleration is the lower signal-to-noise ratio since the sample is split into virtual layers in the spatial encoding.^34^ However, the UF IR-SPICY can be run multiple times during the conventional and IR-SPICY experiment to partially compensate sensitivity loss.^34,42^ Furthermore, as the full 2D *T*_1_–*T*_1ρ_ can be measured by a single scan by the UF IR-SPICY, it is much easier to use modern nuclear-spin hyperpolarization methods to enhance sensitivity of the experiment than in the experiments performed with the conventional or IR-SPICY approaches. For example, typically the polarization build-up in dissolution dynamic nuclear polarization (dDNP)^28^ method takes from tens of minutes to hours, and repeating the hyperpolarization procedure tens of times is practically impossible. Furthermore, typically the polarization level varies quite a lot in repeated hyperpolarization processes. As hyperpolarization increases experimental sensitivity up to five orders of magnitude, hyperpolarized single-scan UF IR-SPICY experiments may enable the measurement of substances with much smaller concentrations.

In principle, spatial encoding used in the UF IR-SPICY experiment requires that the sample be homogeneous in the spatial encoding region. However, as shown above, the effects of inhomogeneous spin density and coil sensitivity profile can be compensated satisfactorily in postprocessing using a 1D image of the sample, leading to *T*_1_ values that are in good agreement with the reference experiments. Even in that case, the UF IR-SPICY experiments require that *T*_1_ times are uniform in the spatial encoding region.

A technical detail that needs to be considered in the analysis of spatially encoded *T*_1_ data is that typically images must be presented in magnitude mode due to phase distortions, and therefore, the negative part of the IR curve is mirrored to the positive intensities, as seen in Figures 2h and 4c. Since the signal changes fastest at the beginning of the IR curve, it might be useful to sample the initial part of the IR curve more densely. This is especially important in the case of multicomponent *T*_1_ relaxation to probe the rapidly relaxing component. In the case of the UF IR-SPICY experiment, linear *T*_1_ data sampling can be changed, e.g., to logarithmic sampling by using nonlinear frequency sweep in the chirp inversion pulse.^67^

## Conclusions

This work introduced two novel and efficient *T*_1_–*T*_1ρ_ correlation experiments: the IR-SPICY and UF IR-SPICY. The first one employs the single-scan acquisition of the *T*_1ρ_ data by the spin-lock cycle (SPICY).^61^ The second, ultrafast (UF) experiment utilizes spatial encoding for *T*_1_ IR dimension so that the whole 2D *T*_1_–*T*_1ρ_ correlation can be observed in a single scan, only in a couple of seconds. Since *T*_1_ relaxation reflects the high-frequency motions and *T*_1ρ_ low-frequency motions, the methods correlate two regions of molecular motions. The methods were demonstrated by a water immersed silica gel sample and a hydrogel sample containing collagen and CS, the main components of articular cartilage. As a result, three distinct components were observed in the silica–water sample indicating pore-located, bulk, and exchanging water and in the hydrogel indicating bulk water, collagen-bound water, and CS-bound water. Both methods shorten the experiment time remarkably as compared to the hypothetical experiments carried out by the conventional approach probing both *T*_1_ and *T*_1ρ_ dimensions point by point. In the case of the IR-SPICY and UF IR-SPICY experiments, the time reduction is one to two and two to three orders of magnitude, respectively. This allows the study of samples that are changing or degrading over time. The single-scan nature of the UF IR-SPICY also significantly facilitates the use of nuclear-spin hyperpolarization methods in *T*_1_–*T*_1ρ_ correlation experiments, potentially boosting sensitivity up to five orders of magnitude, which allow the detection of low concentration molecules even at the physiological level.^46^

## Supporting Information Available

Pulse sequence codes for IR-SPICY and UF IR-SPICY (PDF)
