# Supplementary material for: Ultrafast T1–T1ρ NMR for Correlating Different Motional Regimes of Molecules
Source: Anal Chem. 2024 Oct 9;96(42):16534–42. doi: 10.1021/acs.analchem.4c00513 (PMC11503516; doi:10.1021/acs.analchem.4c00513)
Supplement: Supplementary file 1 — ac4c00513_si_001.pdf [file ac4c00513_si_001.pdf]

Supporting Information

Ultrafast T_1-$T_{1\rho}$ NMR for correlating different motional regimes of molecules

Katja Tolkkinen,* Otto Mankinen, Sarah E. Mailhiot, Ville-Veikko Telkki*

NMR Research Unit, Faculty of Science, University of Oulu, P.O. Box 3000, 90014 Oulu, Finland

*Corresponding authors. Email: katja.tolkkinen@oulu.fi, ville-veikko.telkki@oulu.fi

Table of contents

1. IR-SPICY pulse sequence S3

2. UF IR-SPICY pulse sequence S5

1. IR-SPICY pulse sequence

```
#include<Avance.incl>
#include<Grad.incl>
#include<De.incl>

define delay digtime
"digtime=dw*td"
"p2=2*p1"
"d23 = (20u+p1+d6)+(11*d6*l4)+(4u+dw*l2)*l4"
"d22=3*d6+d21/2"

1  ze                                                    ;zero memory
2  d1                                                    ;relaxation delay
  (p2 pl1 ph12):f1                                       ;180 degree inversion pulse
  vd                                                     ;variable recovery delay
  (rde1 sync de1 adc ph31 syrec) (1u 1u ph30:r):f1 (de)  ;start receiving
  10u setrtp1^5
  10u sytra
  (p1 pl1 ph1):f1                                        ;90 degree pulse, set power level pl1

  d6 pl26:f1                                             ;set power level for cw pulse
  d22                                                    ;delay to center spin echo
3  (d6 cpd1 ph2):f1                                      ;apply cw pulse
  d20                                                    ;wait while cw is on
  d6 do:f1                                               ;stop cw pulse
  d8
  (p2 pl1 ph2):f1                                        ;180 degree pulse, set power level pl1
  d8
  d6 pl26:f1
  (d6 cpd1 ph2):f1                                       ;apply cw pulse
  d20                                                    ;wait while cw is on
  d6 do:f1                                               ;stop cw pulse
  d6 syrec
  d6 setrtp1|5
  d6 setrtp1|0
```

```
  d21                                                    ;acquire signal
  d6 setrtp1^0
  d6 setrtp1^5
  d6 sytra
  lo to 3 times l4                                       ;SPICY loop, l4 = number of echoes
  10u setrtp1^5
  rcyc=2
  100m
  1s wr #0 if #0 ivd                                     ;write data, increment vd
  lo to 1 times td1                                      ;IR loop
  exit
ph12 =0 0 2 2                  ;chirp
ph1  =0 0 2 2 1 1 3 3          ;90 degree pulse
ph2  =1 3 1 3 0 2 0 2          ;180 degree pulse
ph30 =0                        ;reference
ph31 =0 0 2 2 1 1 3 3

;pl1 : f1 channel - power level for pulse
;pl26: f1 channel - power level for cw pulse, 10 % of maximum is safe
;p1 : f1 channel -  high power pulse
;d1 : relaxation delay; 1-5 * T1
;ns: number of scans
;d6: min delay, 5 microseconds
;l4 : number of echoes
;cpdprg1: choose witchey
;vdlist: variable delay list
;d20: constant cw pulse time
;d8: delay before and after 180 pulse
;d22: delay to center spin echo
;d21: off time delay to acquire data (minimize to prevent T2 decay and diffusion effects during acquisition)
```

2. UF IR-SPICY pulse sequence

```
#include<Avance.incl>

#include<Grad.incl>

#include<De.incl>


define delay digtime

                    "digtime=dw*td"

                    "d15 = 66*dw"

                    "d23 = d2 - de"

                    "d17=0.5*p1"

                    "p2=2*p1"

                    "d3= digtime/2 + d23 +de - d16/2"

                    "d4 = d7/2 - p2/2 - p1/2 - 2*d16 - d3 - d15"

                    "d5 = d7/2 - p2/2 - d16 - d23 - de - digtime/2 -d15"


200u UNBLKGRAMP


start,      100u

start1,     200u pl1:f1                                  ;power level on ch1


d1                                                       ;relaxation delay

d16 grad {(0)|(0)|step(cnst23,10)}                       ;ramp up, delay d

d2

 p3:sp0:f1 ph5                                           ;frequency sweep inversion pulse

 d16 grad {(0)|(0)|(cnst23)-step(cnst23,10)}             ;ramp down, delay d

d2 pl1:f1

p1 ph1                                                   ;90 degree high power pulse

echo,

d6 pl26:f1                                               ;set power level for cw pulse

(d6 cpd1 ph2):f1                                         ;apply cw pulse

d20                                                      ;wait while cw is on

d6 do:f1                                                 ;stop cw pulse

d8

 (p2 pl1 ph2):f1                                         ;180 pulse

 d8
```

```
d6 pl26:f1                                          ;set power level for f1,
 (d6 cpd1 ph2):f1                                   ;apply cw pulse
d20                                                 ;wait while cw is on
 d6 do:f1                                           ;stop cw pulse
d17                                                 ;echo time compensation
d16 grad {(0)|(0)|step(cnst20,10)}                  ;gradient ramp up
d3                                                  ;delay for read dephase
d15                                                 ;delay due to digital filtering compensation
d16 grad {(0)|(0)|(cnst20)-step(cnst20,10)}         ;gradient ramp down
 d4                                                 ;echo time compensation
(p2 pl1 ph2):f1                                     ;180 pulse
d5                                                  ;echo time compensation
d16 grad {(0)|(0)|step(cnst20,10)}                  ;gradient ramp up
d15                                                 ;delay due to digital filtering compensation
d15                                                 ;delay due to digital filtering compensation
d23                                                 ;frequency of acquisition
ACQ_START(ph30,ph31)                                ;start receiving
digtime DWELL_GEN:f1                                ;start acquisition
d2 st                                               ;gradient stabilization delay, move to next boot block in
memory
d16 grad {(0)|(0)|(cnst20)-step(cnst20,10)}         ;gradient ramp down
d5                                                   ;echo time compensation
 (p2 pl1 ph2):f1                                    ;180 degree pulse
d16 grad {(0)|(0)|step(cnst20,10)}                  ;gradient ramp up
d3                                                  ;delay for read dephase
d15                                                 ;delay due to digital filtering compensation
d16 grad {(0)|(0)|(cnst20)-step(cnst20,10)}         ;gradient ramp down
d4
lo to echo times nbl

500u eoscnp                                          ;acquisition
100u ipp2 ipp31 ipp1                                 ;phase increment
lo to start times ns                                 ;in memory acquisition loop
100u wr #0 if #0
10u
exit
```

```
ph5= 0 0 2 2                    ;chirp pulse
ph1=  0 2 0 2 1 3 1 3           ;90 degree pulse
ph2=  3 3 1 1 0 0 2 2           ;180 degree pulse and cw pulse
ph30= 0                         ;reference phase used by aq start
ph31= 3 1 3 1 0 2 0 2           ;receive

;pl1 : f1 channel - power level for pulse
;pl26: f1 channel - power level for cw pulse, 10 % of maximum power is safe
;p1 : f1 channel -  90 degree high power pulse
;p3 : f1 channel -  180 degree high power pulse
;d1 : relaxation delay (1-5 * T1)
;d2 : gradient stabilization delay (50 us)
;d4 : TE pad delay
;d5 : TE pad delay
;d16: gradient ramp delay
;d15: delay due to digital filtering compensation
;d7 : spin echo time
;d8 : spin echo time
;d20 : constant cw pulse time
;d6 : cw set time (5 us)
```
